# High canola meal inclusion in gestation and lactation sow diets with *Saccharomyces cerevisiae* product on reproductive performance, milk composition, and nutrient digestibility of sow and litter performance

**DOI:** 10.1093/tas/txaf113

**Published:** 2025-08-20

**Authors:** Xiaoxiao Zhang, Debora Muratori Holanda, Anna Rogiewicz, Elijah G Kiarie, Chengbo Yang, Charles Martin Nyachoti

**Affiliations:** Department of Animal Science, University of Manitoba, Winnipeg, Manitoba, R3T 2N2, Canada; Department of Animal Science, University of Manitoba, Winnipeg, Manitoba, R3T 2N2, Canada; Department of Animal Science, University of Manitoba, Winnipeg, Manitoba, R3T 2N2, Canada; Department of Animal Biosciences, University of Guelph, Guelph, Ontario, N1G 2W1, Canada; Department of Animal Science, University of Manitoba, Winnipeg, Manitoba, R3T 2N2, Canada; Department of Animal Science, University of Manitoba, Winnipeg, Manitoba, R3T 2N2, Canada

**Keywords:** canola meal, digestibility, milk, piglets, probiotic, sows

## Abstract

The objective of this study was to assess how sow and litter performance and nutrient utilization were affected by dietary probiotic supplementation in gestation and lactation diets that contained high levels of canola meal. Seventy-five sows were allotted to one of three treatment diets, starting on d 80 of gestation. The experimental diets included a control diet (**CTRL**) composed of corn and soybean meal, or a modified CTRL diet where soybean meal was substituted with 300 g/kg of canola meal, provided either with (**CCM-P**) or without (**CCM**) *Saccharomyces cerevisiae* product supplementation. On d 80 and d 111 of pregnancy, as well as on d 1 and d 21 following farrowing, the sow body weight (**BW**) and backfat thickness were recorded. Piglet weights were measured on d 1 and d 19 after birth. Milk and blood samples from sows were collected on d 1 and d 19 post-farrowing to measure nutrient composition. Additionally, fecal samples were gathered on d 110 of gestation and d 19 of lactation to analyze apparent total tract digestibility (**ATTD**) with titanium dioxide as an indicator. Data were analyzed through the PROC MIXED procedure in SAS 9.4, following a randomized complete block design. Results indicated that the inclusion of CCM in sow diets had no significant effect on sow or litter growth performance, as well as plasma urea N levels. However, in contrast to gestating sows fed the CTRL diet, those fed the CCM diet had lower (*P* < 0.05) ATTD of gross energy, dry matter, and crude protein. In contrast, the CCM-P diet led to increased (*P* < 0.05) ATTD of phosphorus and tended to increase (*P* = 0.08) ATTD of calcium relative to the CCM group. Furthermore, lactating sows fed CCM diets demonstrated higher (*P* < 0.05) ATTD of neutral detergent fiber (**NDF**) compared to the CTRL group. Milk fat content was significantly greater (*P* < 0.05) in sows consuming CCM diets than those fed the CTRL diet. In conclusion, incorporating 300 g/kg canola meal into sow diets during late gestation and lactation maintained similar reproductive and litter performance compared to the control diet but negatively impacted nutrient digestibility in late gestating sows. Supplementing the canola meal diet with *S. cerevisiae* product improved phosphorus digestibility and milk fat content, suggesting that probiotics may mitigate some negative effects of canola meal in sow nutrition.

## Teaser

The inclusion of 300 g/kg canola meal, combined with *Saccharomyces cerevisiae* product supplementation, in diets for sows during late gestation and lactation, effectively supported both maternal and piglet growth, yielding performance outcomes comparable to those achieved with soybean meal.

## INTRODUCTION

Feed represents the most significant cost in swine production, making it critical to identify cost-effective alternative protein sources (Boumans et al., 2022). Canola meal, a secondary product derived from oil extraction of canola seeds, is a valuable alternative to soybean meal due to its availability, affordability, favorable crude protein (**CP**) content and well-balanced amino acid (**AA**) composition (Khajali and Slominski, 2012; Adewole et al., 2016). However, the inclusion level of canola meal in swine diets is limited due to anti-nutritional factors, such as glucosinolates, which negatively affect nutrient digestibility and animal performance. Over time, plant breeding and processing advancements have significantly reduced glucosinolates content in canola meal, improving its safety and applicability in animal nutrition (Canola Council of Canada, 2024). Consequently, the adoption of low-glucosinolate canola meal in swine diets has increased over time.

However, the total dietary fiber content in canola meal is approximately 33.80%, which is notably higher than that of soybean meal (21.80%), can negatively impact nutrient digestibility by physically interfering with enzyme activity and nutrient absorption (Agyekum and Nyachoti, 2017). Therefore, when formulating swine diets with canola meal, it is essential to consider standardized ileal digestible (**SID**), AA contents and net energy (**NE**) values. It has been showed that adult pigs, particularly gestating sows, exhibit enhanced energy utilization from the dietary fiber abundant in canola meal, compared to younger pigs (Noblet et al., 2013). For gestating sows, high-fiber diets help manage feed intake, preventing excessive fat accumulation that can lead to insulin resistance, metabolic imbalances, and impaired lactation (Jo and Kim, 2023). Dietary fiber in gestation diets without modifying daily net energy intake has been shown to enhance voluntary feed intake during lactation, however, this increase may not be sufficient to fulfill the nutrient requirements for milk production or to maintain ideal body condition in highly prolific sows (Jo and Kim, 2023). Several studies have explored the use of canola meal in sow diets on sow reproductive performance and their litter performance. For example, King et al. (2001) reported no adverse effects on feed intake, body weight, or piglet growth performance when 200 g of canola meal was included per kg of diet for lactating sows. Likewise, Velayudhan & Nyachoti (2017) reported no notable negative impacts on sow or piglet performance when canola meal was included at 300 g/kg in diets for lactating sows, although digestibility of energy and nutrients was slightly reduced. These findings showed that canola meal could be a feasible alternative to soybean meal in swine diets. However, strategies to mitigate its potential limitations, such as reduced nutrients digestibility, need to be further investigated.

Studies have indicated that supplementation with *Saccharomyces cerevisiae* (***S. cerevisiae***) enhances gut health, nutrient utilization, and animal performance through mechanisms such as immunomodulation, modulation of gut microbiota, enhancement of digestive enzyme activity, and optimization of the gastrointestinal environment (Rauch and Lynch, 2010; Shen et al., 2011). In gestating sows, the addition of yeast has been associated with improved maternal protein utilization, as indicated by lower plasma urea nitrogen levels. It has also been linked to enhanced litter performance, including greater weaning weights and increased average daily gain (Shen et al., 2009). One previous study showed that sows fed with 5 × 10^10^ CFU/kg *S. cerevisiae* from late gestation until weaning had a reduced farrowing duration and backfat losses during lactation and increased feed intake of sows and feed conversion ratio of creep feed in suckling pigs (Sun et al., 2022). These findings underscore the potential of *S. cerevisiae* product as a beneficial feed additive for sows. To the best of our knowledge, however, no in vivo studies have clearly examined how canola meal and *S. cerevisiae* product interact to affect sow reproductive function, highlighting a crucial area for further study. Accordingly, this study aimed to evaluate the impact of high canola meal inclusion, with or without probiotic supplementation, in late-gestating and lactating sows on reproductive performance and nutrient digestibility, as well as suckling piglet growth.

## MATERIALS AND METHODS

### Animals and Experimental Design

The experimental procedures received approval from the University of Manitoba Animal Care Committee (AC11687), and all sows and piglets were managed in accordance with the Canadian Council on Animal Care (CCAC, 2009) guidelines.

This study was conducted at the Glenlea Research Station, University of Manitoba (Winnipeg, MB, Canada). A total of seventy-five gestating sows (TN70, Topigs Norsvin, Oak Bluff, MB, Canada) were included on d 80 of gestation, averaging a parity of 2.7 ± 0.33 and an initial backfat thickness of 15.2 ± 0.58 mm. A randomized full block design was used to allocate sows at random to one of three dietary groups. The trial was carried out across three consecutive farrowing groups, with farrowing occurring every 3 wk. Each treatment group included 25 sows per farrowing group.

From d 80 until d 111 of pregnancy, sows were kept in group housing in gestation stalls individual pens. On d 111 of pregnancy, sows were washed and transferred to the farrowing room, where each crate (2.10 × 0.69 m) was equipped with an individual feeder and water dispenser to ensure unrestricted access to feed and water for both sows and piglets. Each pen was equipped with a heat lamp and a mat to provide warmth for piglets. In the farrowing room, the temperature was gradually reduced from 38 °C to 31 °C by the time of weaning. To balance litter sizes, cross-fostering was carried out within treatment groups within 48 hours after birth, ensuring each sow nursed approximately 12 to 13 piglets. At around 21 d of age, piglets were weaned from the sow.

### Experimental Diets

For both lactating and gestating sows, the experimental diets were prepared using standardized ileal digestible (**SID**) AA and net energy (**NE**) values (Table 1). The nutritional needs for gestating sows as stated by the NRC (2012) were met or exceeded in the formulation of all diets. The lactation diets were formulated for piglets who were predicted to gain 230 g of weight per day and sows that had an average post-farrowing body weight of 210 kg. The SID AA values for soybean meal and canola meal were sourced from previous studies (González-Vega and Stein, 2012; Velayudhan et al., 2019). The study included three dietary treatments: 1) **CTRL**, corn-soybean meal-based diet; 2) **CCM,** corn- 300 g/kg canola meal-based diet; 3) **CCM-P,** CCM diet supplemented with *S. cerevisiae* product (Actisaf® Sc 47, *Saccharomyces cerevisiae*: CNCM I-4407, 10^10^ CFU/g, Phileo Lesaffre Animal Care, France). Gestation diets include 250 mg/kg of *S. cerevisiae* product, while lactation diets contain 500 mg/kg of *S. cerevisiae* product based on the manufacturer’s recommendation. Additionally, titanium dioxide was incorporated at 3.0 g/kg as an indigestible marker.

**Table 1.** Ingredient composition and analyzed nutrient content of experimental diets (as-fed basis)

Item	Diet^1^			
	Gestation^2^		Lactation^3^	
	CTRL	CCM	CTRL	CCM
Ingredient, g/kg				
Corn	720.2	625.0	707.8	618.8
Canola meal	-	300.0	-	300.0
Soybean meal	220.0	-	225.0	-
Vegetable oils	23.0	44.0	25.5	45.0
Limestone	13.0	12.5	11.6	11.5
Monocalcium phosphate	9.8	3.0	15.0	7.8
Salt	4.0	4.0	4.7	4.7
Vitamin-mineral premix^4^	10.0	10.0	10.0	10.0
_L-_ Lysine	-	1.5	0.4	1.8
_L-_Tryptophan	-	-	-	0.1
_L-_Valine	-	-	-	0.3
Calculated composition				
Gross energy, kcal/kg	4,025	4,215	3,985	4,170
Net energy, kcal/kg	2,604	2,603	2,568	2,568
Crude protein, %	17.00	17.03	16.60	16.60
Calcium, %	0.74	0.73	0.76	0.76
Total phosphorus, %	0.55	0.56	0.63	0.65
STTD P, %	0.30	0.30	0.39	0.39
Analyzed composition				
Gross energy, kcal/kg	4,073	4,225	4,064	4,244
Crude protein, %	17.20	17.70	17.50	17.40
Crude fat, %	5.54	7.77	5.85	7.63
Acid detergent fiber, %	2.95	7.00	2.58	6.45
Neutral detergent fiber, %	8.07	12.98	6.42	12.58
Calcium, %	0.64	0.66	0.66	0.63
Total phosphorus, %	0.55	0.58	0.64	0.64
Total glucosinolates (μmol/g)	0.05	0.64	0.07	0.75

^1^Experimental diets consisted of CTRL, corn-soybean meal-based diet; 2) CCM, corn-300 g/kg canola meal-based diet; 3) CCM-P, CCM diet supplemented with *Saccharomyces cerevisiae* product (Actisaf® Sc 47). 250 mg/kg of *Saccharomyces cerevisiae* product in gestation diets, 500 mg/kg of *Saccharomyces. cerevisiae* product in lactation diets.

^2^As-fed basis. All gestation diets were formulated to contain 2.60 Mcal/kg of NE with 0.80%, 0.28%, 0.56%, and 0.17% standardized ileal digestible Lys, Met, Thr, and Trp, respectively, and 0.73% and 0.30% Ca and standardized total tract digestible (STTD) P, respectively. Values were calculated based on values for standardized ileal digestibility of AA in corn and crystalline AA published by NRC (2012), and standardized ileal digestibility values for AA in soybean meal and canola meal were from González-Vega & Stein, (2012) and Velayudhan et al. (2019).

^3^All lactation diets were formulated to contain 2.56 Mcal/kg of NE with 0.84%, 0.26%, 0.55%, and 0.17% standardized ileal digestible Lys, Met, Thr, and Trp, respectively, and 0.76% and 0.39% Ca and STTD P, respectively.

^4^Supplied the following per kg of finished gestation diets: vitamin A, 4400 IU; vitamin D, 880 IU; vitamin E, 48 IU; vitamin K, 0.6 mg; choline, 1.37 g; pantothenic acid, 13 mg; riboflavin, 4.13 mg; folic acid, 1.43 mg; niacin, 11 mg; thiamin, 1.1 mg; vitamin B6, 1.1 mg; biotin, 0.21 mg; vitamin B12, 16 μg, Cu, 11 mg as copper sulfate; Zn, 110 mg as zinc oxide; Fe, 88 mg as ferrous sulfate; Mn, 27.5 mg as manganese sulfate; I, 0.15 mg as potassium iodate; Se, 0.16 mg as sodium selenite. Supplied the following per kg of finished lactation diets: vitamin A, 2200 IU; vitamin D, 880 IU; vitamin E, 48 IU; vitamin K, 0.6 mg; choline, 1.37 g; pantothenic acid, 13 mg; riboflavin, 4.13 mg; folic acid, 1.43 mg; niacin, 11 mg; thiamin, 1.1 mg; vitamin B6, 1.1 mg; biotin, 0.21 mg; vitamin B12, 16 μg, Cu, 22 mg as copper sulfate; Zn, 110 mg as zinc oxide; Fe, 88 mg as ferrous sulfate; Mn, 27.5 mg as manganese sulfate; I, 0.15 mg as potassium iodate; Se, 0.16 mg as sodium selenite.

### Growth and Reproductive Performance

Sows were provided with 3.0 kg/day of the gestation diet from d 80 until d 111 of gestation, followed by 3.0 kg/day of the lactation diet from d 111 until farrowing. After farrowing, sows were offered feed three times daily at 0700, 1100, and 1500 h. The amount of lactation feed was gradually increased by approximately 0.5 kg/day until d 6, after which feed was made available ad libitum until weaning on d 21. Throughout both gestation and lactation, the amount of feed supplied, and feed leftover were weighed daily. The average daily feed intake (**ADFI**) was calculated as the total feed provided minus any feed refusals.

On d 80 and d 111 of pregnancy, body weight (**BW**) and backfat thickness were measured, along with additional assessments on the first day of lactation (farrowing) and at weaning (d 21 of lactation). An a-mode ultrasonic device (Renco Lean-Meater series 12, Renco Corporation, Minneapolis, MN, USA) was used to measure backfat thickness. Measurements were taken at the 10th rib, 6 cm lateral to the midline. Values from both sides were averaged to obtain a single backfat measurement, following the method described by Wang et al. (2008) to measure changes in sow body condition. Estrus detection was performed after weaning to assess the interval from weaning to estrus.

Litter size, including the total number of piglets born, born alive, mummified, and weaned per sow, was recorded. Litters were weighed on the first day of lactation (d 1). To account for age differences due to varying farrowing dates, all piglets were weighed again on d 19 post-farrowing to determine litter weight gain.

### Blood Collection and Plasma Urea Nitrogen Analysis

On d 1 and d 19 post-farrowing, blood samples were obtained from sows by performing venipuncture on the jugular vein and collected into 10 mL sodium heparinized vacutainer tubes (BD Vacutainer®, Franklin Lakes, NJ, USA) with sampling conducted 2 h after the morning feeding. Following collection, blood samples were centrifuged at 3,000 × g for 15 minutes at 4 °C. Before being subjected to additional examination, the collected plasma was placed in plastic screw-cap vials and kept at −80 °C. Plasma was transported to Manitoba Veterinary Diagnostic Services for measurement of plasma urea N concentrations utilizing a VITROS 250 Chemistry System (Ortho-Clinical Diagnostics Inc., Raritan, Rochester, NY).

### Colostrum and Milk Collection and Analysis

On the day of farrowing, 50 mL of colostrum was collected, and an additional 50 mL of milk was obtained on d 19 post-farrowing. Sows were injected with 1 mL oxytocin (Rafter 8 Products Inc., Calgary, AB, Canada) before sample collection to facilitate milk release. Fresh colostrum and milk samples were manually collected from all functional teats, thoroughly mixed, and sent to Horizon Lab Ltd. (Winnipeg, MB, Canada) for analysis of crude fat, crude protein, and lactose composition using Fourier transform infrared spectroscopy with the CombiFoss 6000 system (Foss Electric, Denmark).

### Fecal Samples Collection and Digestibility Analysis

On d 110 of pregnancy and d 19 post-farrowing, fecal samples were obtained from all sows through grab sampling via rectal palpation. Prior to additional analysis, the obtained samples were kept at −20 °C. Fecal samples were ground into a fine powder and dried at 55 °C in a forced-air oven before to chemical examination.

Both diet and fecal samples were ground to a fine consistency for dry matter (**DM**), gross energy (**GE**), CP, Ca, P and neutral detergent fiber (**NDF**) composition analysis. The content of acid detergent fiber (**ADF**), crude fat and glucosinolates content in diets were also determined. Dry matter was analyzed following AOAC (method 934.01; 2006), while GE was quantified using an adiabatic bomb calorimeter (model 6400, Parr Instrument, Moline, IL, 2005) with benzoic acid as a calibration standard. Crude protein was estimated based on nitrogen content multiplied by 6.25, whereas nitrogen content was determined through the combustion method (method 990.03; AOAC, 2006) using a LECO N analyzer (model CNS-2000; LECO Corp., St. Joseph, MI). Calcium and phosphorus concentrations were assessed after ashing and processed according to AOAC (2006; method 985.01), followed by analysis with a Varian inductively coupled plasma mass spectrometer (Varian Inc., Palo Alto, CA). Crude fat concentrations were analyzed in diets following AOAC (method 920.39; 2006). Neutral detergent fiber and ADF were measured using the Ankom 200 Fiber Analyzer (Ankom Technology, Fairport, NY), following the method described by Van Soest et al. (1991). Titanium dioxide content was examined based on the method of Lomer et al. (2000) and analyzed using an inductively coupled plasma spectrometer (Vista-MPX; Varian Canada Inc., Mississauga, ON, CA). Glucosinolate levels in the diets were evaluated according to the procedures outlined by Slominski and Campbell (1987).

### Calculations and Statistical Analysis

The equation was used to calculate the apparent total tract digestibility (**ATTD**) of nutrients:


$$\begin{array}{l} ATTD\left(\%\right)=100-\left\{\left[\left(Nd/Nf\right)\times \left(Tf/Td\right)\right]\times 100\right\} \end{array}$$

In the equation, Nd represents the concentration of energy or nutrients in fecal samples, Nf denotes the energy or nutrient levels in feed, Tf corresponds to the titanium dioxide concentration in feed, and Td refers to the titanium dioxide concentration in fecal samples.

The MIXED procedure in SAS (SAS Inst. Inc., Cary, NC, USA) was used to analyze the data, applying a randomized complete block design. In the model, treatment was considered a fixed effect, while farrowing group and block were included as random effects. To account for variability due to seasonal and management factors, farrowing group was set as a random variable. Each sow or litter served as the experimental unit. Initial backfat thickness on d 80 of pregnancy was incorporated as a covariate for BW and backfat thickness analysis. Covariates were retained in the model only if their effects were significant (*P *< 0.10); otherwise, they were excluded.

To assess normality and variance homogeneity, residuals were examined using diagnostic plots generated from the model. The Shapiro-Wilk test and Levene’s test were applied to evaluate residual normality and homogeneity, respectively. Outliers were excluded if standardized residuals exceeded ± 3 standard deviations. Least square means were analyzed, and differences were considered significant at *P* < 0.05, while trends were identified for values between 0.05 and 0.10.

## RESULTS

### Sow Performance

No dietary effect (*P* > 0.10) was found on sow BW or backfat thickness during the entire experimental period (Table 2). However, sows fed the CCM diet had a tendency (*P* = 0.09) for lower BW gain from d 80 to d 111 of pregnancy compared to those fed the CTRL diet. No difference (*P* > 0.10) in ADFI in lactation period was found among sows fed the different experimental diets. Dietary treatment had no effect (*P* > 0.10) on the wean to estrus interval of sows.

**Table 2.** Effect of dietary canola meal inclusion and probiotic in sow diets on sow performance during late gestation and lactation

Item	Diet^1^			SEM	*P*-value
	CTRL	CCM	CCM-P		
Parity	2.83	2.96	2.60	0.325	0.731
Sow body weight, kg					
d 80 of gestation	267.3	264.7	262.4	8.55	0.924
d 111 of gestation	301.9	297.7	294.6	8.61	0.717
d 1 post-farrowing	280.2	280.8	276.4	4.09	0.698
d 21post-farrowing	249.9	250.0	247.5	6.89	0.944
Gestation gain^2^	30.26	24.38	26.19	1.96	0.085
Lactation loss^3^	30.18	26.77	29.35	5.00	0.535
Sow backfat thickness, mm					
d 80 of gestation^4^	15.18	14.99	15.35	0.409	0.818
d 111 of gestation	15.53	15.84	15.84	0.542	0.826
d 21 post-farrowing	13.31	14.42	13.31	0.636	0.183
Gestation gain	0.32	0.71	0.59	0.542	0.830
Lactation loss^5^	1.70	1.40	2.29	0.514	0.458
Gestation ADFI, kg/d	2.98	2.96	2.96	0.012	0.166
Lactation ADFI, kg/d	5.99	5.72	5.65	0.291	0.162
Wean to estrus interval, d	4.22	4.13	4.28	0.223	0.880

^1^Experimental diets consisted of CTRL, corn-soybean meal-based diet; 2) CCM, corn-300 g/kg -based diet; 3) CCM-P, CCM diet supplemented with *Saccharomyces*. *cerevisiae* product (Actisaf® Sc 47). 250 mg/kg of *Saccharomyces cerevisiae* product in gestation diets, 500 mg/kg of *Saccharomyces cerevisiae* product in lactation diets.

^2^Gestation bodyweight or backfat thickness gain was calculated as the difference between sow bodyweight at d 80 and d 111 of gestation.

^3^Lactation bodyweight gain was calculated as the difference between sow body weight at d 1 and d 21 of post-farrowing.

^4^Sow initial backfat thickness at d 80 of pregnancy was used as a covariate in the statistical model for BW and backfat thickness data analysis.

^5^Lactation backfat thickness loss was calculated as the difference between d 111 of gestation and d 21 of post-farrowing.

### Litter Performance

As shown in Table 3, there were no effects (*P* > 0.10) of sow dietary treatment on number of total piglets born, piglets born live, born dead and mummies. No difference (*P* > 0.10) on the number of piglets after cross-fostering, as intended, or on d19 post-farrowing. Sow dietary treatments had no effect (*P* > 0.10) on the survival rate from farrowing to weaning. In addition, the average body weight of piglets on farrowing and d19 post-farrowing were not influenced (*P* > 0.10) by dietary treatment.

**Table 3.** Effect of dietary canola meal inclusion and probiotic in sow diets on reproductive performance at farrowing and litter performance during the suckling period

Item	Diet^1^			SEM	*P*-value
	CTRL	CCM	CCM-P		
Litter size^2^					
Total born	16.69	15.24	16.53	0.817	0.236
Born alive	15.53	14.09	15.32	0.629	0.140
Stillborn	1.08	0.96	1.40	0.309	0.573
Mummified	0.17	0.16	0.20	0.086	0.938
After cross-fostering	14.34	13.43	13.90	0.554	0.115
Weaned	12.82	12.19	12.42	0.300	0.193
Piglet survival pre-weaning,^3^ %	90.11	88.85	89.27	2.203	0.891
Litter weight, kg					
d 1	20.65	18.92	19.64	1.543	0.136
d 19	76.33	72.21	72.93	3.779	0.331
Daily weight gain,^4^ kg/d	2.93	2.80	2.81	0.140	0.521

^1^Experimental diets consisted of CTRL, corn-soybean meal-based diet; 2) CCM, corn-300 g/kg -based diet; 3) CCM-P, CCM diet supplemented with *Saccharomyces cerevisiae* product (Actisaf® Sc 47). 250 mg/kg of *Saccharomyces cerevisiae* product in gestation diets, 500 mg/kg of *Saccharomyces cerevisiae* product in lactation diets.

^2^Litter size, the number of piglets per litter.

^3^Piglet survival pre-weaning (%) = (the number of weaned piglets/the number of piglets after cross fostering) × 100.

^4^Daily weight gain = (litter weight on d 19—litter weight on d 1)/19.

### Milk Composition and Plasma Urea N

The nutrient composition of sow colostrum and milk, including crude fat, crude protein, lactose, and oligosaccharides, is presented in Table 4. No significant differences (*P* > 0.10) were observed among treatments for these components. There was no difference (*P* > 0.10) on milk crude protein and lactose contents among sows fed different diets. However, sows fed the CCM-P diet had a significantly higher (*P* < 0.05) crude fat value than sows fed the CTRL and CCM diet on d 19 post-farrowing.

**Table 4.** Effect of dietary canola meal inclusion and probiotic on colostrum and milk composition on d 19 post-farrowing

Item	Diet^1^			SEM	*P*-value
	CTRL	CCM	CCM-P		
Colostrum composition, g/kg					
Crude fat	7.32	8.08	7.40	0.446	0.422
Crude protein	6.56	6.41	6.89	0.399	0.683
Lactose	5.60	5.59	5.59	0.095	0.988
Milk composition, g/kg					
Crude fat	7.43^b^	7.48^b^	8.24^a^	0.309	0.014
Crude protein	4.61	4.61	4.79	0.084	0.213
Lactose	7.18	7.12	7.11	0.068	0.509

^a,b^Within a row, means with different superscripts differ (*P* < 0.05).

^1^Experimental diets consisted of CTRL, corn-soybean meal-based diet; 2) CCM, corn-300 g/kg -based diet; 3) CCM-P, CCM diet supplemented with *Saccharomyces cerevisiae* product (Actisaf® Sc 47). 250 mg/kg of *Saccharomyces cerevisiae* product in gestation diets, 500 mg/kg of *Saccharomyces cerevisiae* product in lactation diets.

Sows fed the CCM diet showed a tendency (*P* = 0.09) for lower plasma urea N levels on farrowing day compared to those fed the CTRL diet, but the addition of probiotics to the CCM-P diet did not affect (*P* > 0.10) the plasma urea N level relative to the CTRL diet as shown in Table 5. No difference (*P* > 0.10) was found in plasma urea N levels of sows fed the CTRL, CCM or CCM-P diets on d 19 post-farrowing.

**Table 5.** Effect of dietary canola meal inclusion and probiotic on plasma urea nitrogen of sows at farrowing and on d 19 post-farrowing

Item	Diet^1^			SEM	*P*-value
	CTRL	CCM	CCM-P		
Plasma urea N, mmol/L					
Farrowing day	3.83	3.34	3.68	0.164	0.089^2^
d 19 post-farrowing	4.58	4.27	4.44	0.143	0.274

^1^Experimental diets consisted of CTRL, corn-soybean meal-based diet; 2) CCM, corn-300 g/kg -based diet; 3) CCM-P, CCM diet supplemented with *Saccharomyces cerevisiae* product (Actisaf® Sc 47). 250 mg/kg of *Saccharomyces cerevisiae* product in gestation diets, 500 mg/kg of *Saccharomyces cerevisiae* product in lactation diets.

^2^The tendency (*P* = 0.09) for lower plasma urea N at farrowing day was observed only between CCM and CTRL.

### Apparent Total Tract Digestibility of Energy and Nutrients

In Table 6, the energy and nutrients digestibility in gestating sows were shown. Sows receiving CCM and CCM-P diets exhibited lower (*P* < 0.05) ATTD values for DM, GE, and CP compared to those fed the CTRL diet. Gestating sows in the CCM-P group tended to have higher Ca digestibility than those in the CCM group (*P* = 0.08), while no difference (*P* > 0.10) was observed between the CCM and CTRL groups. While no significant differences (*P* > 0.10) were observed in the ATTD of P between sows fed the CTRL and CCM diets, the probiotic supplementation in the CCM-P diet significantly improved (*P* < 0.05) the ATTD of P relative to the CCM diet. Nevertheless, the ATTD of Ca and P in sows fed the CCM-P diet was comparable (*P* > 0.10) to that in the CTRL group.

**Table 6.** Effect of dietary canola meal inclusion and probiotic on apparent total tract digestibility (ATTD) coefficients of nutrients and energy in gestation sows on d 110 of gestation

Item	Diet^1^			SEM	*P*-value
	CTRL	CCM	CCM-P		
Dry matter, %	85.87^a^	82.60^b^	83.13^b^	0.63	< 0.001
Gross energy, %	85.83^a^	83.34^b^	83.40^b^	0.68	< 0.001
Crude protein, %	86.47^a^	81.51^b^	82.59^b^	0.65	< 0.001
Calcium^2^, %	34.63	31.47	37.75	1.89	0.085
Phosphorus, %	31.96^a^	27.23^b^	33.52^a^	2.04	0.011
NDF^3^, %	52.35	52.19	52.56	2.35	0.991

^a,b^Within a row, means with different superscripts differ (*P* < 0.05).

^1^Experimental diets consisted of CTRL, corn-soybean meal-based diet; 2) CCM, corn-300 g/kg canola meal-based diet; 3) CCM-P, CCM diet supplemented with *Saccharomyces cerevisiae* product (Actisaf® Sc 47). 250 mg/kg of *Saccharomyces cerevisiae* product in gestation diets, 500 mg/kg of *cerevisiae* product in lactation diets.

^2^A tendency for higher (*P* = 0.08) calcium digestibility was observed in the CCM-P group compared with the CCM group, and no difference was observed between CCM-P and CTRL groups.

^3^NDF, neutral detergent fiber.

Table 7 showed that the ATTD coefficients for energy and nutrients in sows fed different dietary treatments during the lactation period. No dietary treatment effects (*P* > 0.10) were observed on the ATTD of GE, CP, Ca or P. However, the addition of probiotic to the CCM-P diet tended to decrease (*P* = 0.07) the ATTD of DM in comparison to sows fed the CTRL diet, while no difference was found between the CCM and CTRL groups. Additionally, sows consuming canola meal-based diets (CCM and CCM-P) showed a significantly higher (*P* < 0.05) ATTD of NDF than those on the CTRL diet.

**Table 7.** Effect of dietary canola meal inclusion and probiotic on apparent total tract digestibility (ATTD) coefficients of nutrients and energy in lactation sows on d 19 post-farrowing

Item	Diet^1^			SEM	*P*-value
	CTRL	CCM	CCM-P		
Dry matter^2^, %	83.57	82.57	82.19	0.57	0.073
Gross energy, %	83.86	83.69	82.83	0.46	0.209
Crude protein, %	84.46	83.47	83.28	0.49	0.141
Calcium, %	30.14	30.56	30.97	1.90	0.923
Phosphorus, %	34.48	32.30	36.75	1.49	0.113
NDF^3^, %	43.27^a^	54.72^b^	54.21^b^	2.24	<0.001

^a, b^Within a row, means with different superscripts differ (*P* < 0.05).

^1^Experimental diets consisted of CTRL, corn-soybean meal-based diet; 2) CCM, corn-300 g/kg canola meal-based diet; 3) CCM-P, CCM diet supplemented with Saccharomyces cerevisiae product (Actisaf® Sc 47). 250 mg/kg of Saccharomyces cerevisiae product in gestation diets, 500 mg/kg of cerevisiae product in lactation diets.

^2^The tendency (*P* = 0.07) for lower dry matter digestibility was observed only between CCM-P and CTRL; no difference was shown between CCM and CTRL.

^3^NDF, neutral detergent fiber.

## DISCUSSION

This study investigated the effects of including 300 g/kg of canola meal in sow diets during late gestation and lactation, with or without probiotic supplementation. Overall, the inclusion of canola meal did not compromise sow reproductive outcomes, litter performance, milk composition, and nitrogen metabolism, except for some reductions in energy and protein digestibility during late gestation. While digestibility of some nutrients was reduced, particularly during late gestation, probiotic supplementation partially alleviated this effect by improving phosphorus digestibility. These findings suggest that canola meal is a potential substitute to replace soybean meal as a protein source in sow nutrition when combined with dietary strategies to optimize nutrient digestibility.

Previous studies (Baidoo et al., 1986; McIntosh et al., 1986) have reported that the inclusion of canola meal in swine diets impaired growth performance, likely due to diet formulations based primarily on CP content and digestible energy (**DE**) values. The DE system often overestimates the energy values of diets high in protein or fiber (Velayudhan et al., 2015). In contrast, the NE system offers a more precise assessment of energy utilization compared to other energy evaluation methods, making it more effective to use canola meal in animal diets without negatively influencing animal performance (Velayudhan et al., 2015; Agyekum and Nyachoti, 2017). It has been suggested that canola meal may be included at levels of up to 200g/kg in diets for lactating sows without negatively affecting reproductive performance or litter growth performance when formulated diets based on NE system (King et al., 2001). The results of sow reproductive performance and litter growth performance in the current study align with more recent research on lactating sows (Velayudhan and Nyachoti, 2017), wherein it was observed that inclusion of up to 300 g/kg canola meal in lactation diet did not change body weight during lactation or weaning to estrus interval when diets were formulated according to SID AA and NE content.

In the current study, sows fed with 300 g/kg canola meal had a similar ADFI during the entire experimental period compared to sows fed with soybean meal diets. This may be partially explained by the higher crude fat levels in CSCM-based diets than CTRL diets (7.77% vs. 5.54% during gestation; 7.63% vs. 5.85% during lactation), which could have improved feed palatability. Dietary fat enhances aroma, texture, and mouthfeel, all of which are known to stimulate voluntary feed intake in pigs (Varona et al., 2021). The improved palatability may have helped mitigate negative effects of high dietary fiber on feed intake. Additionally, dietary fiber has a higher heat increment due to its fermentation in the hindgut and associated increase in metabolic heat, while dietary fat has the lowest heat increment among macronutrients (Schoenherr et al., 1989). Therefore, the higher fat content in the CSCM-based diets may have helped reduce the additional heat production typically associated with fiber-rich formulations, thereby supporting more efficient energy utilization and maintaining feed intake. However, in previous studies, an increased level of canola meal in the diet of weaned or growing pigs led to a decrease in ADFI, closely related to the metabolic effects of glucosinolates, which disrupt iodine metabolism, affect thyroid gland function, and thereby influence animal performance (Mullan et al., 2000; Parr et al., 2015). As demonstrated by Bell (1993) and Mawson et al. (1994), growing pigs may tolerate up to 2.5 μmol/g of total glucosinolates in their diets, while sows can take up to 4.0 μmol/g without experiencing any negative effects on their reproductive performance. However, in the current study, the glucosinolate concentration in canola meal was 2.4 μmol/g and the glucosinolate in gestation and lactation diets were 0.6 μmol/g and 0.7 μmol/g, respectively, therefore, the low glucosinolates may explain why sows fed diets containing high levels of canola meal did not experience a decrease in ADFI in this study. Similarly, previous studies demonstrated that diets with glucosinolate concentrations below 2.0 μmol/g do not negatively impact daily feed intake in lactating sows (Schöne et al., 2001; Quiniou et al., 2012). The unaffected ADFI in sows fed canola meal in the current study may also be attributed to increased gut capacity following an adaptation period. Previous research has shown that diets rich in non-starch polysaccharides (**NSP**) or pectin led to the development of larger stomachs and colons in growing pigs, with both volume and empty weight increasing (Jørgensen et al., 1996b). Several studies have observed an increase in feed intake during lactation when high-fiber diets were provided during gestation, likely due to the gastrointestinal tract becoming adapted to the larger volumes of feed associated with high fiber intake (Agyekum et al., 2019; Shang et al., 2019). However, the differences in results may be attributed to variations in dietary fiber types and the length of the adaptation period. Thus, future studies should further explore the long-term effects of high-fiber canola meal diets during both gestation and lactation on gut capacity and feed intake in sows and digestive system adaptation.

In addition, the present experiment showed that litter performance, including litter size at farrowing and weaning, as well as piglet average daily gain, was not affected by dietary treatment. These results are consistent with the findings of Velayudhan and Nyachoti (2017), who similarly reported no adverse effects when 300 g/kg of canola meal was included in sow diets. These results support the hypothesis that canola meal can be used in sow diets to fully replace soybean meal without adverse effects on sow and suckling piglet performance. The lack of effect on litter size at birth may be due to the limited influence of dietary changes during late gestation on the number of piglets born. Although there was a numerical difference (approximately 1.5 piglets) in the number of piglets born alive between the CTRL and CCM groups, this difference was not statistically significant. To further explore whether this variation was influenced by total litter size, we included total born as a covariate in the statistical model. After adjustment, the least square means (± SEM) for piglets born alive were CTRL: 14.61 ± 0.22, CCM: 14.61 ± 0.22, and CCM-P: 14.91 ± 0.22. The treatment effect remained non-significant (*P *> 0.10), while total born as a covariate was highly significant (*P* < 0.001). These results suggest that the variation in the number of piglets born alive was primarily driven by natural variation in total litter size, rather than by a direct effect of dietary treatment. However, litter size at birth is largely determined by ovulation rate, early embryonic survival, and uterine capacity, which are established during early gestation (Bennett and Leymaster, 1989). Additionally, litter size at weaning primarily depends on neonatal survival, which is largely influenced by the piglet’s energy reserves from hepatic and muscular glycogen, as well as intake of colostrum and transitional milk in the first few days after birth (Theil et al., 2014). Piglet average daily gain is largely related to both the quantity and quality of milk provided by the sow, although other factors such as litter size, piglet health, and environmental conditions also contribute to growth (King et al., 1997). In the present study, the absence of dietary effects on litter size at weaning and litter weight gain likely reflects the fact that milk composition was not adversely affected by the inclusion of 300 g/kg of canola meal in the maternal diet. The precise formulation of diets to meet SID amino acid and NE requirements likely ensured adequate lactational nutrient supply, which helped maintain piglet growth and survival despite the higher fiber content and inclusion of 300 g/kg canola meal in the CCM diet. Furthermore, the litter or piglet weights at birth and weaning were not significantly affected by the supplementation of *S. cerevisiae* product in this study. This is in line with other research (Jang et al., 2013; Le Floc′h et al., 2022) that demonstrated that live yeast supplementation in sow diets had no effect on growth performance of their litters. However, yeast culture supplementation during pregnancy and lactation has been shown in some studies to increase litter growth performance (Kim et al., 2008; Liu et al., 2023; Christensen et al., 2024). The tested probiotic in the present study is a live yeast (*S. cerevisiae*), in contrast to yeast culture, which consists of yeast metabolites and cereal grain fermentation byproducts that may provide additional nutrients to enhance litter weight gain. It has also been reported that live yeast increased litter weight gain and average weaned piglets per litter in sows receiving live yeast (Domingos et al., 2021). Furthermore, variations in yeast strain, dosage, and duration of supplementation may influence the degree and consistency of live yeast effects on sow reproductive outcomes (Patterson et al., 2023).

Milk composition is a crucial factor for the growth and development of suckling piglets (Farmer, 2013). The composition and yield of colostrum and milk in sows are influenced by a number of factors, such as the environment, diets, breed, and health state (Amatucci et al., 2022). The current study found no significant difference in milk composition between sows fed canola meal diets and those in the control group, consistent with findings from Velayudhan and Nyachoti (2017). The underlying mechanism is likely due to the diets being formulated with equivalent NE and SID AA contents. Additionally, sow milk production tended to remain stable even when dietary protein and energy levels are slightly deficient (De Bettio et al., 2016). During late gestation, sows allocate energy and AA to fetal and placental growth, fluids and membrane as well as for mammary gland development (Langendijk et al., 2023). In lactation, most of the absorbed nutrients and energy are directed toward milk production and further mammary gland development (Bauman and Bruce Currie, 1980; Hurley et al., 2000). When sows do not receive sufficient nutrients, particularly protein and energy, they break down body tissue protein to maintain milk production (Dourmad et al., 2008). The absence of major differences between the CTRL and CCM groups in milk composition suggests that sows fed the high-fiber canola meal diet maintained adequate nutrient intake during lactation to support milk production without the need for substantial body weight or backfat mobilization, as reflected in the unchanged sow performance indicators.

In the present study, dietary supplementation with *S. cerevisiae* product during lactation increased milk fat concentration at weaning in sows fed canola meal-based diets. Similar findings have been reported with yeast culture, a fermentation-derived product of *S. cerevisiae*, which, when supplemented at 500 or 800 mg/kg from day 30 of gestation, increased colostrum fat and lactose concentrations (Ma et al., 2023). However, not all studies have reported positive effects of yeast supplementation on milk composition. For instance, Jang et al. (2013) observed no improvements in milk composition, including fat, lactose and protein, when live yeast was provided in lactation. One possible explanation for these divergent findings is the substantial difference in yeast dosage: while Jang et al. used live yeast at 10⁷ CFU/g, the present study applied a higher dosage of 10¹⁰ CFU/g at 500 mg/kg during lactation. In the present study, one possible explanation for the increase in milk fat concentration is the enhanced nutrient digestibility observed with live yeast supplementation. By promoting beneficial gut microbiota and stabilizing the gastrointestinal environment, live yeast may improve the breakdown and absorption of dietary fiber, thereby increasing the energy supply required for milk fat synthesis (Kritas et al., 2006). In addition, sows fed canola meal-based diets supplemented with probiotics produced more milk fat compared to those in the CTRL group. While this may be partially attributed to the probiotic effects, another contributing factor could be the higher crude fat content in the CCM-P diets during lactation. Review papers by Pettigrew (1981) and Rosero et al. (2016) summarized that fat-rich diets increase milk fat output in sows by stimulating milk fat synthesis, with additional benefits for piglet growth. However, in this study, neither the higher crude fat levels in the CCM and CCM-P diets nor the inclusion of *S. cerevisiae* product improved the growth performance of suckling piglets. The limited impact of dietary fat on early piglet growth may be explained by the fact that protein and water retention are the primary drivers during this stage (Noblet and Etienne, 1987). It has been shown that dietary fat intake and milk fat output are not major determinants of piglet growth, as evidenced by Neal et al. (1999), who found no significant difference when fat levels were increased from 3% to 9%. Instead, the quantity of milk consumed may play a more critical role during the suckling phase than the actual composition of the milk (Quesnel et al., 2012). This aligns with previous findings showing that increased milk production in sows and greater milk intake by piglets are strongly correlated with piglet daily weight gain (Strathe et al., 2020).

Urea is the primary nitrogenous waste product formed during the breakdown of dietary protein that is either not utilized by the body or results from tissue protein turnover (Weiner et al., 2015). In this study, during gestation, sows fed the CCM diet tended to have lower plasma urea N levels and reduced ATTD of CP. This effect may be attributed to the higher fiber content and the slightly lower AA digestibility compared to soybean meal, which can hinder protein digestion and absorption (Newkirk and Classen, 2002). The decreased digestibility of CP could also result in less nitrogen being absorbed, thereby contributing to reduced plasma urea N levels (Eggum, 1970). However, during lactation, neither the ATTD of CP nor plasma urea N levels were significantly affected by the canola meal diet, indicating that all diets were balanced for AA to the same degree. This stability could be due to the increased protein demand during lactation, which enhances nitrogen utilization efficiency (Kim et al., 2013). Additionally, improved nitrogen recycling and potential adaptations of the gut microbiota to the higher fiber content of the canola meal diet may contribute to these outcomes.

Research on the effects of canola meal on nutrient digestibility shows mixed results, with some indicating reduced energy and nutrient absorption (Velayudhan and Nyachoti, 2017), while others found no negative effects when substituting soybean meal with canola meal (Agyekum et al., 2014; Sanjayan et al., 2014). These discrepancies may arise from variations in factors such as fiber composition, processing methods, and the physiological state of the pigs (Noblet and Shi, 1993; McDougall et al., 1996). Therefore, understanding the impact of canola meal on nutrient digestibility, especially in sow diets, is essential. In the present study, inclusion of 300 g/kg of canola meal during gestation resulted in reduced ATTD of DM, GE and CP. This reduction may be attributed to several interacting factors, particularly the higher crude fat and dietary fiber contents in the canola meal-based diet compared to the control diet. Several studies have demonstrated that dietary fat can enhance nutrient digestibility. For instance, Jørgensen et al. (1996a) reported improved ATTD of CP when pigs were fed diets containing 4%, 8%, and 16% rapeseed oil. Furthermore, pigs fed oil-supplemented diets showed higher ATTD of fat compared to those fed basal diets (Jørgensen et al., 2000). However, Jørgensen and Fernández (2000) observed that at higher fat inclusion levels, fat digestibility becomes independent of dietary fat content. While the true ileal and total tract digestibility of fat may remain constant at higher inclusion levels (Kil et al., 2010), increased fiber intake can lead to greater bile secretion and mucosal turnover, thereby increasing endogenous fat excretion and reducing ATTD values. This may help explain why, in the current study, the crude fat level of 7.7% did not result in improved energy or protein digestibility. In addition, the increased dietary fiber content in this study may be the cause of the declines in energy and crude protein digestibility values of late gestating sows fed diets containing 300 g/kg of canola meal. Although the increased fiber did not negatively affect growth indicators in this trial, the reduction in nutrient digestibility coefficients aligned with previous findings, where lactating sows fed diets containing 15% to 30% canola meal from d 115 of gestation until weaning exhibited similar outcomes (Velayudhan and Nyachoti, 2017). Likewise, inclusion 200 g/kg canola meal to replace soybean meal in weaned pig diets decreased the ATTD coefficients of DM, GE and CP (Wang et al., 2017). The reduced ATTD of CP suggested that canola meal supplied less digestible AA, likely due to the presence of hulls in canola meal, which contain 150 g/kg CP but are challenging to digest (Khajali and Slominski, 2012).

In the current study, lactating sows fed diets containing 300 g/kg canola meal showed no differences in nutrients and energy digestibility, which is consistent with findings by Velayudhan et al., (2018), where high-fiber canola meal diets did not affect energy and nutrient digestibility coefficients. This lack of impact could be attributed to the sow ability to adapt over time, allowing them to more efficiently utilize the high dietary fiber (Jørgensen et al., 1996b). However, lactating sows fed canola meal-containing diets demonstrated increased ATTD of NDF compared to those fed the CTRL diet in the present study. This may be partially explained by the higher NDF levels in the canola meal diets. Additionally, progressive adaptation to the canola meal diet over time may have contributed to improved fiber utilization during the later stages of the current study. Despite using similar diet formulations with 300 g/kg of canola meal during lactation, Velayudhan and Nyachoti (2017)  did not observe any improvement in NDF digestibility, which may be due to differences in the duration of dietary exposure. Given the potential for ingredient effects to accumulate across reproductive cycles, future research could explore how long-term inclusion of canola meal from early gestation over multiple reproductive cycles affects sow nutrient metabolism and lifetime productivity.

Supplementation of canola meal-based diets with *S. cerevisiae* product improved the ATTD of P during gestation. This improvement is likely linked to the enzymatic activity stimulated by the yeast, particularly phytase, which can hydrolyze phytate complexes in plant-based ingredients and enhance the release of bound minerals (Kaur et al., 2007). Previous studies have also reported similar effects of yeast products on phosphorus availability. For instance, inclusion of live yeast in sow diets during gestation and lactation improved phosphorus digestibility in nursery pigs (Lu et al., 2019). Fermentation of dietary fiber has been suggested to increase intestinal phosphorus availability in pigs (Metzler and Mosenthin, 2008), and improved P digestibility has been observed in broilers and growing pigs supplemented with yeast culture (Gao et al., 2008; Kim et al., 2014). However, no improvement in Ca and P digestibility was observed during lactation in the present study. This differential response may be attributed to physiological differences between gestation and lactation. During lactation, sows consume significantly more feed, which may accelerate gastrointestinal transit time and reduce the contact time required for effective enzymatic hydrolysis of phytate complexes (De Bettio et al., 2016). As a result, the addition of *S. cerevisiae* product may enhance the nutritional value of canola meal by mitigating its negative impact on phosphorus digestibility during gestation, thereby improving its feasibility as a sustainable alternative to soybean meal in sow diets.

## CONCLUSION

In conclusion, canola meal at 300 g/kg can effectively replace soybean meal in gestating and lactating sow diets. The inclusion of canola meal negatively influenced the apparent total tract digestibility of energy and nutrients during gestation but without compromising sow or litter performance. The supplementation of *Saccharomyces cerevisiae* product alleviated the negative impact of canola meal on phosphorus digestibility in gestating sows and increased milk fat content at weaning. While these findings highlight the potential of canola meal as a sustainable protein source, the reduction in energy and protein digestibility was only observed during gestation, but not during lactation, suggesting that sows may gradually adapt to canola meal with repeated use. Therefore, further studies are needed to determine whether long-term feeding across multiple cycles can mitigate the initial negative effects observed during gestation.
